# The adipokinetic hormone family in Chrysomeloidea: structural and functional considerations *

**DOI:** 10.3897/zookeys.157.1433

**Published:** 2011-12-21

**Authors:** Gerd Gäde, Heather G. Marco

**Affiliations:** 1 Zoology Department, University of Cape Town, Rondebosch, South Africa

**Keywords:** Cerambycidae, Chrysomelidae, adipokinetic hormone, structure elucidation, mass spectrometry, phylogenetic relatedness

## Abstract

The presented work is a hybrid of an overview and an original research paper on peptides belonging to the adipokinetic hormone (AKH) family that are present in the corpora cardiaca of Chrysomeloidea. First, we introduce the AKH/red pigment-concentrating hormone (RPCH) peptide family. Second, we collate the available primary sequence data on AKH peptides in Cerambycidae and Chrysomelidae, and we present new sequencing data (from previously unstudied species) obtained by liquid-chromatography coupled with ion trap electrospray ionisation mass spectrometry. Our expanded data set encompasses the primary structure of AKHs from seven species of Cerambycidae and three species of Chrysomelidae. All of these species synthesise the octapeptide code-named Peram-CAH-I (pGlu-Val-Asn-Phe-Ser-Pro-Asn-Trp amide). Whereas this is the sole AKH peptide in Cerambycidae, Chrysomelidae demonstrate a probable event of AKH gene duplication, thereby giving rise to an additional AKH. This second AKH peptide may be either Emppe-AKH (pGlu-Val-Asn-Phe-Thr-Pro-Asn-Trp amide) or Peram-CAH-II (pGlu-Leu-Thr-Phe-Thr-Pro-Asn-Trp amide). The peptide distribution and structural data suggest that both families are closely related and that Peram-CAH-I is the ancestral peptide. We hypothesise on the molecular evolution of Emppe-AKH and Peram-CAH-II from the ancestral peptide due to nonsynonymous missense single nucleotide polymorphism in the nucleotide coding sequence of prepro-AKH. Finally, we review the biological significance of the AKH peptides as hyperprolinaemic hormones in Chrysomeloidea, i.e. they cause an increase in the circulating concentration of proline. The mobilisation of proline has been demonstrated during flight in both cerambycid and chrysomelid beetles.

## 1. The adipokinetic hormone (AKH)/red pigment-concentrating hormone (RPCH) family of peptides: general background information

Peptides belonging to the AKH/ RPCH family are produced in neurosecretory cells of either the corpora cardiaca (CC) of insects or of the X-organ cells in the eyestalks of decapod crustaceans (see reviews by Gäde 1997; Gäde et al. 1997b; Gäde and Marco 2006). In crustaceans, the classical effect of RPCH is the concentrating movement of pigment granules in integument cells causing a blanching appearance of the whole body (see review by Rao 2001). In insects, AKHs classically control the mobilisation of fuels for active muscular exertion, such as flight, swimming or running (see reviews by Gäde 2004; Gäde and Marco 2009). Such metabolites can be lipids, carbohydrates or the amino acid proline, and the neuropeptides involved in this action are then generally referred to as adipokinetic (lipid-mobilising), hypertrehalosaemic (increase in the level of the haemolymph sugar, trehalose) or hyperprolinaemic (increase of haemolymph proline levels). After release of the neuropeptides from the CC into the haemolymph during flight for example, they bind to G-protein-coupled receptors, which are known from a limited number of insects to date, and in turn activate either a triacylglycerol lipase or a glycogen phosphorylase in the fat body via diverse second messenger systems (see review by Gäde and Auerswald 2003). Whereas only two structurally different octapeptides are known from Crustacea (Fernlund and Josefsson 1972; Christie et al. 2008; Marco and Gäde 2010), about 50 isoforms are known from various insect species (Gäde 2009; Gäde and Simek 2010; Marco et al. 2011). These peptides have the following common structural characteristics: a chain length of 8–10 amino acids, post-translationally modified termini (the N-terminus contains a pyroglutamate residue and the C-terminus is carboxyamidated), at least two aromatic amino acids (at position 4 and 8), a glycine residue at position 9, and largely, but not exclusively, the peptides are uncharged with only 4 of the 50 odd members having a charged asparagine residue at position 7 (Gäde 2009).

The primary structure of peptides from the AKH/RPCH family have been used as an additional data set to aid in the construction of phylogenies in insect orders (Gäde 1989; Gäde et al. 1994, 1997a, 2003a,b; Gäde and Simek 2010; Kodrík et al. 2010). Despite the few character states, these structures supported, for example, a high-level phylogeny for Odonata (Gäde and Marco 2005; Gäde et al. 2011) when used in conjunction with published ideas on Odonata phylogenies (see, for example, Bechly 1996; Misof 2000; Ware et al. 2007; Dumont et al. 2010).

In the present study we focus on the AKHs from certain beetle families, namely the superfamily Chrysomeloidea. Here, we review the previously published data on AKHs in this superfamily and add hitherto unpublished structural data on AKHs of more species from the two subfamilies, Chrysomelidae and Cerambycidae, with the aim of drawing preliminary conclusions on the putative value of this data set for scientific speculation on relatedness of these families. We also review the current knowledge on the function of AKHs in both subfamilies with respect to the control of substrate availability, especially the amino acid proline.

## 2. The AKH/RPCH peptide family in Chrysomeloidea

The order Coleoptera constitutes the most species-rich taxon on Earth with approximately 350 000 described species and comprises, therefore, about 40 % of all insect species and about 25 % of all animal species (Hammond 1992; Grove and Stork 2000). About 75 % of beetle species are phytophagous and within these “Phytophaga” two superfamilies, Curculionoidea and Chrysomeloidea, are especially diverse and total about 130 000 species. The Chrysomeloidea is usually divided in five families (Crowson 1981) of which the Cerambycidae (longhorn beetles) and Chrysomelidae (leaf beetles) are the most radiated ones with about 35 000 species each. In the current work, we have compiled an AKH data base using seven cerambycid and three chrysomelid species.

### 2.1. Insects included in the current data set

The cerambycids *Ceroplesis capensis* Linnaeus, 1764 and *Promeces longipes* (Olivier, 1795) were collected in September 2009 and 2010 in the Cedarberg close to Clanwilliam, South Africa, while the eucalyptus borer *Phoracantha recurva* Newman, 1840 was found in the cellar of a house in Cape Town, S. Africa after the purchase and storage of logs from eucalyptus trees which were apparently infested with borer larvae and pupae (2009). The chrysomelid *Chrysolina kuesteri* (Helliesen, 1912) was collected at the beginning of November 2007 in a vineyard in Frickenhausen, Germany. The cerambycid *Leptura maculata* Poda, 1761 and the chrysomelid *Chrysolina fastuosa* Scopoli, 1763 were collected in June 2011 in the forest adjacent to the campus of the University of Konstanz in Germany. For collection details of the cerambycids *Ceroplesis thunbergii* Fåhraeus, 1872, *Phryneta spinator* (Fabricius, 1792) and *Morimus funereus* Mulsant, 1863, see Gäde and Auerswald (2000; 2001), and for the chrysomelid *Leptinotarsa decemlineata* Say, 1824, see Gäde and Kellner (1989) and Gäde (1999).

### 2.2 Structure elucidation and diversity of AKHs

We followed standard laboratory procedures to identify and obtain the primary structure of AKHs from the CC of Chrysomeloidea species. In all cases, the CC were dissected into 80% methanol and extracts were prepared by sonification according to Gäde et al. (1984). AKH peptides from *Ceroplesis thunbergii*, *Phryneta spinator*, *Leptinotarsa decemlineata* and *Morimus funereus* were then purified by reversed-phase high performance liquid chromatography (RP-HPLC) as outlined in Gäde (1985) and identified in biological assays; finally, material from active fractions were sequenced by the Edman degradation technique (see Gäde and Kellner 1989; Gäde and Auerswald 2000).

**Figure 1.**
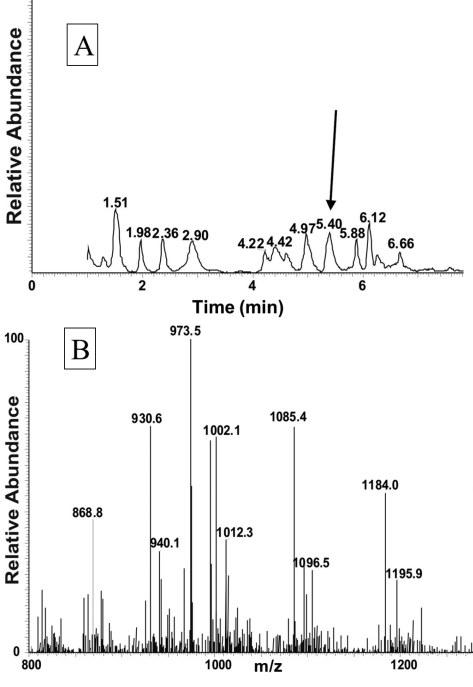
LC-MS analysis of an extract from the corpora cardiaca of *Ceroplesis capensis*. Material from the CC of the cerambycid beetle, *Ceroplesis capensis* was extracted with 80% methanol and analysed by LC-MS. **A** The total ion chromatogram **B** A full scan positive electrospray ionisation (ESI) mass spectrum of the peak shown in (**A**) with a retention time of 5.40 min.

**Figure 2.**
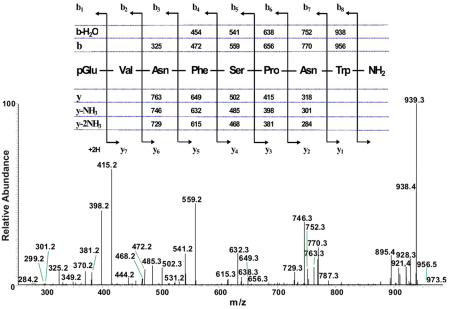
The collision-induced dissociation ESI mass spectrum of an AKH peptide in *Ceroplesis capensis*, and its deduced amino acid sequence. The CID MS^2^-ESI spectrum of the ion MH^+^ (*m*/*z* 973.5) in Fig. 1B. The inset shows the assigned peptide sequence (corresponding to that of Peram-CAH-I), together with the theoretical calculated masses for b-type and y-type diagnostic fragment ions which are observed in the MS^2^ mass spectrum.

**Figure 3.**
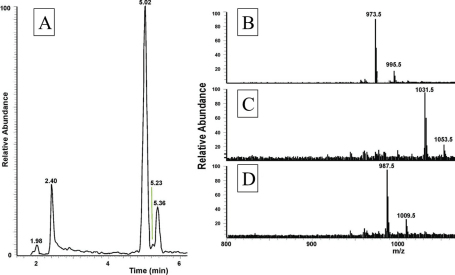
LC-MS analysis of an extract from the corpora cardiaca of *Chrysolina kuesteri*. Material from the CC of the chrysomelid beetle, *Chrysolina kuesteri* was extracted with 80% methanol and analysed by LC-MS. (A) The total ion chromatogram. Full scan positive ESI mass spectra of the peaks shown in (**A**) with a retention time of 5.02 min (**B**), 5.23 min (**C**) and 5.36 min (**D**).

**Figure 4.**
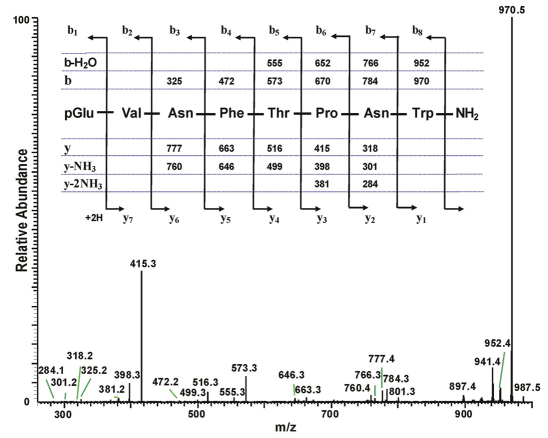
The collision-induced dissociation ESI mass spectrum of an AKH peptide in *Chrysolina kuesteri*, and its deduced amino acid sequence. The CID MS^2^ ESI spectrum of the ion MH^+^ at *m*/z 987.5 (see Fig. 3D). The inset shows the sequence of the assigned peptide (corresponding to that of Emppe-AKH), together with the theoretical calculated masses for b-type and y-type diagnostic fragment ions which are observed in the MS^2^ mass spectrum.

**Figure 5.**
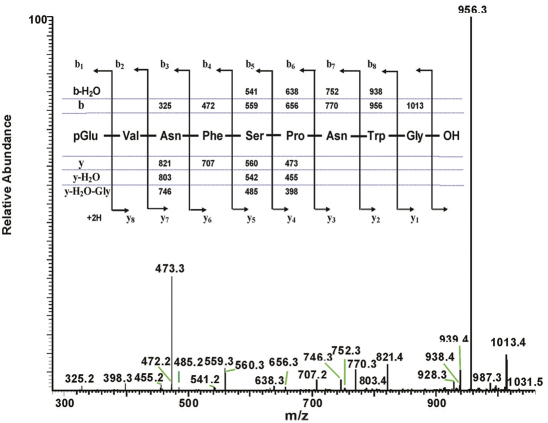
The collision-induced dissociation ESI mass spectrum of an additional putative AKH peptide in *Chrysolina kuesteri*, and its deduced amino acid sequence. The CID MS^2^ ESI spectrum of the ion MH^+^ at *m*/z 1031.5 (see Fig. 3C). The inset shows the sequence of the assigned peptide (corresponding to an intermediate processing form of Peram-CAH-I, i.e. a non-amidated nonapeptide with a Gly residue at position 9). Also shown are the theoretical calculated masses for b-type and y-type diagnostic fragment ions which are observed in the MS^2^ mass spectrum.

For the remaining species: *Ceroplesis capensis*, *Promeces longipes*, *Phoracantha recurva*, *Leptura maculata*, *Chrysolina kuesteri* and *Chrysolina fastuosa*, peptide material in the CC extracts were analysed directly by liquid chromatography-mass spectrometry (LC-MS) using a quadropole ion trap mass spectrometer equipped with an electrospray ionisation (ESI) source (instruments and methods as described in detail elsewhere (see Kodrík et al. 2010). This strategy has been employed successfully for a number of other insect species (see for example Gäde et al. 2003, 2009; Gäde and Simek 2010), and produces clean, easily interpretable data for which only a fraction of gland material is required when compared with enzymatic degradation techniques. For the current study, the mass spectrometry results are exemplarily illustrated from a CC extract of a cerambycid, *Ceroplesis capensis* (Figs 1–2), and of a chrysomelid, *Chrysolina kuesteri* (Figs 3–5).

The total ion chromatogram of about 0.5 pair equivalent of CC from *Ceroplesis capensis* showed one peak of interest at a retention time of 5.40 min (Fig. 1A); this peak displays inter alia an ion with highest abundance at *m*/*z* 973.5 for the [M + H]^+^ ion (Fig. 1B). The primary sequence of the peptide was deduced from the MS^2^ spectrum obtained by collision-induced dissociation (CID) of the precursor shown in Fig. 2. The characteristic y-type and b-type product ions, in conjunction with diagnostic ions (i.e. y-NH_3_, y-2NH_3_ and b-H_2_O) enabled the assignment of an [M + H]^+^ ion as a member of the AKH family (see inset in Fig. 2). Although the alignment of the first two amino acids cannot be inferred from the recorded MS^2^ spectrum, we know from the general signature structure of AKHs that pyroglutamate is typically at the N-terminus, which leaves only a valine residue at position two to fill the gap of the measured 99 atomic mass units. Thus, the sequence of the AKH synthesised in the CC of *Ceroplesis capensis* is assigned as pGlu-Val-Asn-Phe-Ser-Pro-Asn-Trp amide which is identical to that of Peram-CAH-I (*Periplaneta americana*-cardioacceleratory hormone-I), which was found for the first time in the cockroach *Periplaneta americana* (Linnaeus, 1758) (Scarborough et al. 1984; Witten et al. 1984).

The total ion chromatogram of about 0.8 pair equivalent of CC from *Chrysolina kuesteri* show three peaks of interest at retention times of 5.02 min (highest abundance), 5.23 min (lowest abundance) and 5.36 min (intermediate abundance) (Fig. 3A); these peaks display ions at *m*/*z* 973.5, 1031.5 and 987.5, respectively for the [M + H]^+^ ions (Figs 3B-D). The MS^2^ spectra obtained by CID of the respective precursors were recorded. Because the spectrum for [M + H]^+^ at *m*/*z* 973.4 was almost identical to the one for *Ceroplesis capensis* (Fig. 2), the data are not illustrated here, and the underlying AKH was easily assigned as Peram-CAH-I. The MS^2^ spectrum for [M + H]^+^ at *m*/*z* 987.5 is given in Fig. 4; the interpretation was straightforward, and a peptide with the primary structure of pGlu-Val-Asn-Phe-Thr-Pro-Asn-Trp amide was assigned, which had previously been found in the CC of the mantid *Empusa pennata* (Thunberg, 1815) called Emppe-AKH (Gäde 1991). The third peptide with [M + H]^+^ at *m*/*z* 1031.5 was assigned as a C-terminal Gly-extended non-amidated form of Peram-CAH-I by its MS^2^ spectrum (Fig. 5).

In the cases detailed above, the assigned amino acid sequence of the native AKHs was confirmed by comparing the retention times, the [M + H]^+^ ions and the respective CID-MS^2^ spectra with those of the appropriate synthetic peptides; chromatographic and mass spectral properties were always identical (data not shown). For use in such confirmatory studies, synthetic peptides Peram-CAH-I and -II (*Periplaneta americana* cardioacceleratory hormone-I and -II) had been purchased from Peninsula Laboratories Inc. (now Bachem Americas Inc., California, USA), while the peptide Emppe-AKH (*Empusa pennata* adipokinetic hormone) had been custom-synthesised by Dr. R. de Milton (Medical School, UCT, South Africa).

### 2.3 Primary sequence diversity of AKHs

Table 1 summarises the structural information of AKHs in chrysomeloid beetles known previously from Edman sequencing (Gäde and Kellner 1989; Gäde and Auerswald 2000), and those (hitherto unpublished) that have recently been elucidated by employing direct analysis of the glandular extract via LC-ESI MS. Although mass spectra of AKHs are only shown for *Ceroplesis capensis* and *Chrysolina kuesteri* in the current work, it should be noted that clear sequencing data were obtained for all chrysomeloid beetles studied in this way. Hence, the current study unambiguously identifies AKHs from the two large families of the Chrysomeloidea via a combined LC-MS approach in comparison with synthetic peptides. These results are very interesting and revealing. First, all AKH members are octapeptides, no decapeptide has been detected yet in any chrysomeloid beetle. In fact, the only decapeptide member sequenced from ANY beetle species to date is the peptide code-named Declu-CC (pGlu-Leu-Asn-Phe-Ser-Pro-Asn-Trp-Gly-Asn amide; *Decapotoma lunata*-CC) which is biosynthesised in the CC of members of blister beetles (Meloidae; Gäde 1995). Second, all investigated chrysomeloid species contain in their CC the peptide Peram-CAH-I (see Table 1). All 7 species of the Cerambycidae, spanning 6 genera, have this peptide as the only AKH member, whereas the three species of Chrysomelidae representing two genera have Peram-CAH-I plus a second AKH peptide (see Table 1). Interestingly, the two species from the same genus (*Chrysolina kuesteri* and *Chrysolina fastuosa*) have the same AKH complement. Additionally, what appears to be an incompletely-processed form of Peram-CAH-I is also detected in the CC of *Chrysolina kuesteri* (see Fig. 5). Whether this non-amidated nonapeptide is biologically active, remains to be determined in the future.

**Table 1.** Adipokinetic hormone sequences in Cerambycidae and Chrysomelidae. Primary sequences of peptides of the adipokinetic hormone family in the corpora cardiaca of various species belonging to the families Cerambycidae and Chrysomelidae.

**Family**	**Species**	**Amino acid sequence**	**Code name of peptide**
Cerambycidae	*Phryneta spinator*	pGlu-Val-Asn-Phe-Ser-Pro-Asn-Trp amide	Peram-CAH-I
	*Ceroplesis thunbergii*	pGlu-Val-Asn-Phe-Ser-Pro-Asn-Trp amide	Peram-CAH-I
	*Ceroplesis capensis*	pGlu-Val-Asn-Phe-Ser-Pro-Asn-Trp amide	Peram-CAH-I
	*Promeces longipes*	pGlu-Val-Asn-Phe-Ser-Pro-Asn-Trp amide	Peram-CAH-I
	*Phoracantha recurva*	pGlu-Val-Asn-Phe-Ser-Pro-Asn-Trp amide	Peram-CAH-I
	*Morimus funereus*	pGlu-Val-Asn-Phe-Ser-Pro-Asn-Trp amide	Peram-CAH-I
	*Leptura maculata*	pGlu-Val-Asn-Phe-Ser-Pro-Asn-Trp amide	Peram-CAH-I
Chrysomelidae	*Leptinotarsa decemlineata*	pGlu-Val-Asn-Phe-Ser-Pro-Asn-Trp amide	Peram-CAH-I
		pGlu-Leu-Thr-Phe-Thr-Pro-Asn-Trp amide	Peram-CAH-II
	*Chrysolina kuesteri*	pGlu-Val-Asn-Phe-Ser-Pro-Asn-Trp amide	Peram-CAH-I
		pGlu-Val-Asn-Phe-Thr-Pro-Asn-Trp amide	Emppe-AKH
	*Chrysolina fastuosa*	pGlu-Val-Asn-Phe-Ser-Pro-Asn-Trp amide	Peram-CAH-I
		pGlu-Val-Asn-Phe-Thr-Pro-Asn-Trp amide	Emppe-AKH

**Figure 6.**
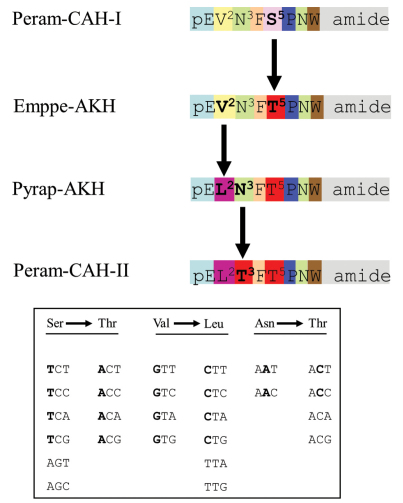
Putative molecular evolution of adipokinetic peptides in chrysomeloid beetles. A proposed scheme of the molecular evolution of adipokinetic peptides to give rise to the different structures observed in investigated chrysomeloid beetles. The inset shows the genetic code for the respective amino acids involved. Differences in amino acids or code nucleotides are given in bold lettering.

From the data in Table 1, it seems that Peram-CAH-I is the ancestral peptide. We propose that the following molecular evolution (Fig. 6) may have occurred by conservative nucleotide substitutions involving nonsynonymous missense single nucleotide polyphemisms (SNPs) in the DNA:

(1) T in position one of the genetic code for serine (S^5^) in Peram-CAH-I was replaced by A to code for threonine (T^5^) in Emppe-AKH;

(2) G in position one of the genetic code for valine (V^2^) in Emppe-AKH was replaced by C to now code for leucine (L^2^) in Pyrap-AKH (*Pyrrhocoris apterus*-adipokinetic hormone);

(3) A in position two of the genetic code for asparagine (N^3^) in Pyrap-AKH was replaced by C and now codes for threonine (T^3^) in Peram-CAH-II (Fig. 6).

The proposed scenario outlined above, appears to be the most likely one. An alternative route may have involved the molecular change from Emppe-AKH to Peram-CAH-II via another intermediate peptide where N^3^ in Emppe-AKH is first changed to T^3^; we find this to be highly unlikely since none of the 50 completely known AKH structures, to date, has the combination of pGlu-Val-Thr at the N-terminus that is required from such an intermediate AKH.

The evolutionary steps proposed in Fig. 6 lead to the peptide Pyrap-AKH, which exists in certain heteropteran and homopteran bugs, in a pamphagid grasshopper and in the red flour beetle, *Tribolium castaneum* (Herbst, 1797) (Gäde 2009). In Chrysomelidae, however, this peptide has not yet been detected. With more chrysomelid beetles to be investigated for their complement of AKH peptides, the proposed hypothesis can be proven or refuted in the future. We would like to emphasise that AKH structures cannot be used as a sole tool for investigation into the phylogenies of Chrysomelidae. Phylogenies have been carried out based on morphological and molecular data (for example, Duckett et al. 2004; Gomez-Zurita et al. 2007) and these two pillars will certainly continue to be central in future investigations, with peptide structure and complement used in a supportive context.

### 2.4. Functional significance of AKH peptides

Previously, we conducted two sets of experiments to determine which substrate is mobilised by the secretion of an AKH in chrysomeloid beetles.

In the first set of experiments, we injected the cerambycid beetles, *Ceroplesis thunbergii* and *Phryneta spinator*, with their own CC extract or a low dose of the identified peptide, Peram-CAH-I, in a synthetic form and measured the concentration of total lipids, total carbohydrates and proline in the haemolymph before injection and 90 min thereafter (Gäde and Auerswald 2000). In both beetles the crude CC extract, as well as the synthetic Peram-CAH-I peptide had a hypertrehalosaemic and hyperprolinaemic effect, i.e. significant increases in the concentration of carbohydrates and proline were measured. In the chrysomelid, *Leptinotarsa decemlineata*, it was shown that injection of conspecific CC extract had a hypertrehalosaemic effect (Gäde and Scheid 1986) and injection of low doses of synthetic endogenous peptides, Peram-CAH-I and -II, caused a significant increase in the proline concentration in the haemolymph (Gäde 1999).

In the second set of experiments we examined the effect of active flight on substrate levels in the haemolymph of chrysomeloid beetles. In this set-up, the beetles were tethered to a flight mill that was modified to allow lift-generating flight; after a 1 min period of such active flying, the beetle was detached from the mill and kept at rest. In both cerambycid species, *Ceroplesis thunbergii* and *Phryneta spinator*, a small but significant decrease in the concentration of carbohydrates was measured in the haemolymph upon 1 min of flight; there was, however no changes measured in the levels of total lipids. In contrast, the proline concentration in these cerambycid species diminished by 20 to 30 % upon 1 min of flight; following an hour of rest subsequent to a short period of active flight, however, the proline concentration returned to pre-flight levels in the haemolymph (Gäde and Auerswald 2000). In the chrysomelid beetle, *Leptinotarsa decemlineata* (Gäde, 1999), we note very similar results, i.e. a decrease in the circulating proline concentration upon 1 min of flight and subsequent restoration of the pre-flight proline level (at rest) within 1 h of rest after the flight episode. The data indicate that AKH peptides are released during flight, which initiate substrate mobilisation for use by the active muscle tissue. During the restorative phase (resting after flight), the flight muscles are no longer active and, consequently, there is a build-up of the substrates in the haemolymph that were released from stored sources in the fat body (see Gäde 2004).

Based on these earlier investigations with chrysomeloid species, we have, thus, shown that proline is used in conjunction with carbohydrates as metabolic fuel to power flight activity (Gäde 1999; Gäde and Auerswald 2000; Gäde and Auerswald 2002). The pathway that involves proline, ultimately relies on the activation of a lipase which is necessary to provide acetyl-CoA units for the formation of proline from alanine (see Gäde and Auerswald 2003). This critical step in the process of supplying proline for metabolic use is under the regulatory control of members of the AKH family (Gäde and Auerswald 2003). We have, however, no information on the respective role played by multiple AKH peptides in fuel mobilisation in an individual insect, e.g. do both AKHs have the same potency and same action in chrysomelid beetles? Further, does the conserved AKH (Peram-CAH-I) in cerambycid beetles hint at a conserved receptor structure? It still remains a biological curiosity as to why certain groups of insects have AKH gene duplication and others not. In conclusion, many more questions remain open in insect studies that may be addressed by additional investigations at different phylogenetic and physiological levels.
